# Different impulse control disorder evolution patterns and white matter microstructural damage in the progression of Parkinson’s disease

**DOI:** 10.3389/fnagi.2023.1260630

**Published:** 2023-12-22

**Authors:** Ling Hu, Changfu Lin, Fabin Lin, Lingling Wang, Zhenzhen Li, Zhijun Cai, Xianghong Liu, Qinyong Ye, Yiwen Wu, Guoen Cai

**Affiliations:** ^1^Department of Neurology, Ganzhou People’s Hospital, Ganzhou, China; ^2^Department of Medicine, Zhangzhou Fifth Hospital, Zhangzhou, China; ^3^Department of Neurosurgery, Fujian Medical University Union Hospital, Fuzhou, China; ^4^Department of Neurology and Institute of Neurology, Ruijin Hospital Affiliated to Shanghai Jiaotong University School of Medicine, Shanghai, China; ^5^Department of Neurology, Fujian Medical University Union Hospital, Fuzhou, China; ^6^Department of Neurology, Fujian Institute of Geriatrics, Fujian Medical University Union Hospital, Fujian Medical University Union Hospital, Fujian, China; ^7^Fujian Key Laboratory of Molecular Neurology, Institute of Neuroscience, Fujian Medical University, Fuzhou, China

**Keywords:** Parkinson’s disease, impulse control disorders, evolution patterns, white matter microstructural, non-motor symptoms

## Abstract

**Background:**

The course of impulse control disorders (ICD) varies in the early stage of Parkinson’s disease (PD).

**Aim:**

We aimed to delineate the association between the evolution pattern of ICD and the progression of PD.

**Methods:**

A total of 321 *de novo* PD patients from the Parkinson’s Progression Markers Initiative database were included. Patients were followed up for a mean of 6.8 years and were classified into different groups according to the evolution patterns of ICD. Disease progression was compared among groups using survival analysis, in which the endpoint was defined as progression to Hoehn and Yahr stage 3 or higher for motor progression and progression to mild cognitive impairment for cognitive decline. In the fourth year of follow-up, four types of ICD evolution patterns were identified: (1) non-ICD-stable (68.2%), a patient who is consistently free of ICD; (2) late-ICD (14.6%), ICD developed during the follow-up of patients; (3) ICD-stable (11.5%), patients showed persistent ICD; and (4) ICD-reversion (5.6%), baseline ICD disappeared during the follow-up of patients with ICD.

**Results:**

The ICD-reversion type shows daily life non-motor symptoms [Movement Disorder Society-Unified Parkinson Disease Rating Scale (MDS-UPDRS) part I], daily life motor symptoms (MDS-UPDRS part II), rapid eye movement sleep behavior disorder, and anxiety symptoms has a greater impact. PD patients with different ICD evolution patterns had different changes in white matter microstructure at the onset of the disease. Those relevant brain regions are involved in ICD and non-motor functions.

**Conclusion:**

Four early ICD evolution patterns are identified in *de novo* PD, with different prognoses and brain white matter microstructural damage patterns, and they may predict motor progression and cognitive decline in PD patients.

## Introduction

Impulse control disorder (ICD) is a non-motor symptom (NMS) in patients with Parkinson’s disease (PD). ICD was described as excessive or harmful urges and behaviors that cause substantial impairment in social and occupational functioning, such as gambling (pathological gambling), hypersexuality (increased pursuit of sex), overeating, shopping too much, dysregulation of dopamine (a tendency to overuse PD medications), and punding (behavior that is repetitive and purposeless) (Joutsa et al., 2012). Between 13 and 37% of Parkinson’s patients have ICD. Jaakkola et al. have found that newly diagnosed PD patients are more likely to develop ICD during follow-up (Jaakkola et al., 2014). PD patients without ICD symptoms at onset could develop ICD over time (Corvol et al., 2018). Studies have shown that PD-ICD is closely related to the use of Da and has a dose-effect relationship (Corvol et al., 2018), suggesting that PD-ICD may represent a different phenotype.

Diffusion tensor imaging (DTI) is a non-invasive form of magnetic resonance imaging (MRI) that clarifies the integrity of the microscopic structure of brain tissue, assesses neuronal connections and the change in microscopic structure, provides a unique quantitative measurement window, and determines the integrity of the brain (Surkont et al., 2021). A previous DTI study, found that the cerebellum, basal ganglia, cortex, and spinal cord projected fiber link interruption are risk factors for ICD (Smith et al., 2016). Yoo et al. found that the PD-ICD group and the PD-non-ICD group had differences in the callous anterior to the brain, left thalamic radiations, partial right thalamus radiations, the right dorsal and posterior cingula, the right internal capsule (genu and posterior limbs), the right superior temporo-occipital lobe, and the right thalamus (Yoo et al., 2015). Research on PD-related ICD is largely based on brain imaging studies, but the evidence is inconclusive. Neuroimaging studies of PD combined with ICD are still mainly involved in abnormal changes in the midbrain-cortex-limbic system-striatal loops. The in-depth study of PD-ICD-related imaging is expected to provide new ideas for the intervention of PD-ICD. White matter microstructural alterations have also been related to neurodegeneration in PD (Atkinson-Clement et al., 2017; Sarasso et al., 2021). Imperiale et al. found extensive disruption of the white matter tracts in patients with Parkinson’s disease-ICD, with increased radial and axial dispersion of the corpus callosum genu and pontine bundle (Imperiale et al., 2018). There may be an important correlation between the changes in white matter microstructure and PD-ICD. In PD patients, the heterogeneity of symptoms may be related to the stage and brain areas affected. Hence, this evolution pattern may have prognostic value.

The present study aimed to identify the different evolutionary types of ICD in PD, analyze the effects of different ICD evolution types on motor and NMSs of PD, and analyze the potential changes of white matter microstructure.

## Results

### Patterns of ICD evolution in PD patients

**Figure 1:**
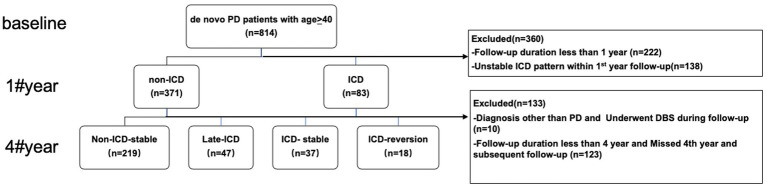
Participants and study flow chart. DBS, deep brain stimulation; PD, Parkinson’s disease; ICD, impulse control disorders.

A total of 321 patients with *de novo* PD were included in this study, with an average follow-up of 6.8 years. As shown in Figure 1, 266 patients had no ICD symptoms at baseline, accounting for 82.8% of patients; 55 patients had ICD at baseline, accounting for 17.1% of patients. In the initial 4 years of follow-up, 37 patients had stable ICD symptoms, accounting for 11.5% of patients, which was the ICD stable group; ICD disappeared in 18 patients, accounting for 5.6% of patients, which was the ICD reversal group, and the average phenotypic transition time was 35.3 months. In addition, 47 PD patients without ICD at baseline developed ICD symptoms, accounting for 14.6% of patients, which was the late ICD group, and the average phenotypic transition time was 38.5 months. However, 219 PD patients still did not have ICD, which was the non-ICD stable group, accounting for 68.2% of patients.

### Clinical characteristics and demographics at baseline of patients from four groups of ICD evolution patterns

A summary of the clinical characteristics and demographics at baseline of patients can be found in Table 1. There were no significant differences between the four groups regarding age, sex, years of schooling, or length of follow-up. Similarly, there was no significant difference in baseline motor function, cognitive function, or index t-score. Patients in the ICD-stable and ICD-reversion groups showed higher MDS-UPDRS part I and II distributions at baseline than patients with non-ICD-stable and late-ICD (*p* = 0.000 < 0.05 and *p* = 0.000 < 0.05, respectively). At baseline, seven patients with H&Y stage ≥3, 37 with mild cognitive impairment (MCI), and 80 with MoCA <26 were identified. The ESS scaled score, Rapid-eye-movement Sleep Behavior Disorder Screening Questionnaire (RBDSQ) scaled score, State Anxiety Inventory (S-AI) scaled score, and Trait Anxiety Inventory (T-AI) scaled score of the non-ICD-stable group were lower than those of the other three groups (*p* = 0.002 < 0.05, *p* = 0.007 < 0.05, *p* = 0.000 < 0.05, and *p* = 0.000 < 0.05, respectively).

**Table 1:** Baseline and demographic characteristics of the study population.

	Non-ICD-stable	Late-ICD	ICD-reversion	ICD-stable	*p*-value
	*n* = 219	*n* = 47	*n* = 18	*n* = 37	
Demographics					
Age, y	62.58 ± 8.81	62.03 ± 8.08	58.41 ± 7.67	62.76 ± 9.09	0.265
Male, *n* (%)	133 (60.7%)	32 (68.1%)	10 (55.6%)	24 (64.9%)	0.721
Years of education, y	15.51 ± 3.43	14.64 ± 4.47	14.28 ± 4.56	15.35 ± 4.81	0.633
Follow-up duration, y	6.68 ± 3.19	5.79 ± 3.8	5.56 ± 2.99	5.86 ± 2.95	0.058
Time to ICD status change, y		3.21 ± 0.85	2.94 ± 0.85		
Motor function					
MDS-UPDRS part I	0.9 ± 1.26	1.32 ± 1.64	3.67 ± 3.27	2.49 ± 2.41	**0.000**
MDS-UPDRS part II	5.54 ± 4.62	5.98 ± 4.37	10.17 ± 6.91	7.97 ± 4.71	**0.000**
MDS-UPDRS part III	20.03 ± 9.16	22.32 ± 9.33	27.44 ± 15.18	20.05 ± 8.93	0.116
H&Y stage (stage 1/stage 2, *n*)	94/120	18/29	4/13	15/20	0.394
H&Y stage ≥ 3 at baseline, *n* (%)	4 (1.8%)	0	1 (5.6%)	2 (5.4%)	0.268
Non-motor function					
ESS scaled score	5.47 ± 3.56	6.66 ± 3.25	8.17 ± 5.96	7.78 ± 4.44	**0.002**
RBDSQ scaled score	3.81 ± 2.66	4.3 ± 2.61	5.89 ± 3.38	4.7 ± 2.92	**0.007**
GDS scaled score	4.36 ± 1.5	4.45 ± 1.87	5.39 ± 1.85	4.76 ± 1.69	0.130
S-AI scaled score	31.79 ± 9.53	33.89 ± 10.35	42 ± 10.94	34.92 ± 10.64	**0.000**
T-AI scaled score	32.91 ± 8.42	34.45 ± 8.98	42.56 ± 9	37 ± 7.96	**0.000**
Cognitive function					
MoCA	26.86 ± 2.44	27.3 ± 2.36	25.67 ± 4.03	25.84 ± 5.07	0.277
MoCA < 26 at baseline	53 (24.2%)	7 (14.9%)	6 (33.3%)	14 (37.8%)	0.087
HVLT-R total recall *t*-test	46.29 ± 10.46	45.49 ± 10.28	43.33 ± 14.56	44.11 ± 11.25	0.505
HVLT-R delay recall	45.63 ± 10.55	44.62 ± 10.14	43.39 ± 14.21	45.76 ± 10.64	0.796
HVLT-R retention	47.64 ± 10.86	47.04 ± 10.28	45.94 ± 14.58	49.51 ± 12.06	0.662
HVLT-R recognition Dis	45.75 ± 10.93	44.19 ± 10.51	42.67 ± 17.23	48.03 ± 9.95	0.315
Index *t*-score					
JLO scaled score	25.47 ± 4.6	25.87 ± 4.12	24.71 ± 4.95	24.81 ± 4.63	0.667
LNS scaled score	10.44 ± 2.63	10.11 ± 2.32	10.67 ± 3.14	10.38 ± 2.82	0.846
SFT t-score	51.58 ± 10.12	50.89 ± 9.55	46.72 ± 16.32	51.11 ± 10.66	0.313
SDMT t-score	44.66 ± 9	44.24 ± 8.78	44.69 ± 12.02	47.58 ± 10.7	0.332
MCI at baseline, *n* (%)	24 (11%)	6 (12.8%)	4 (22.2%)	3 (8.1%)	0.460

Significant *p*-values are shown in bold (*p* < 0.05). Pairwise comparisons were made using Bonferroni correction. ESS, Epworth Sleepiness Scale; GDS, Geriatric Depression Scale; HVLT-R, Hopkins verbal learning test revised; H&Y, Hoehn–Yahr; JLO, judgment of line orientation; LNS, Letter-Number Sequencing; MCI, mild cognitive impairment; MDS-UPDRS, Movement Disorder Society Unified Parkinson’s Disease Rating Scale; MoCA, Montreal Cognitive Assessment; RBDSQ, REM Sleep Behavior Disorder Screening Questionnaire; S-AI, State Anxiety Inventory; SFT, Semantic Fluency Test; SDMT, Symbol Digit Modalities Test; T-AI, Trait Anxiety Inventory.

### The relationship between ICD evolution patterns and non-motor function progression In PD patients

**Figure 2:**
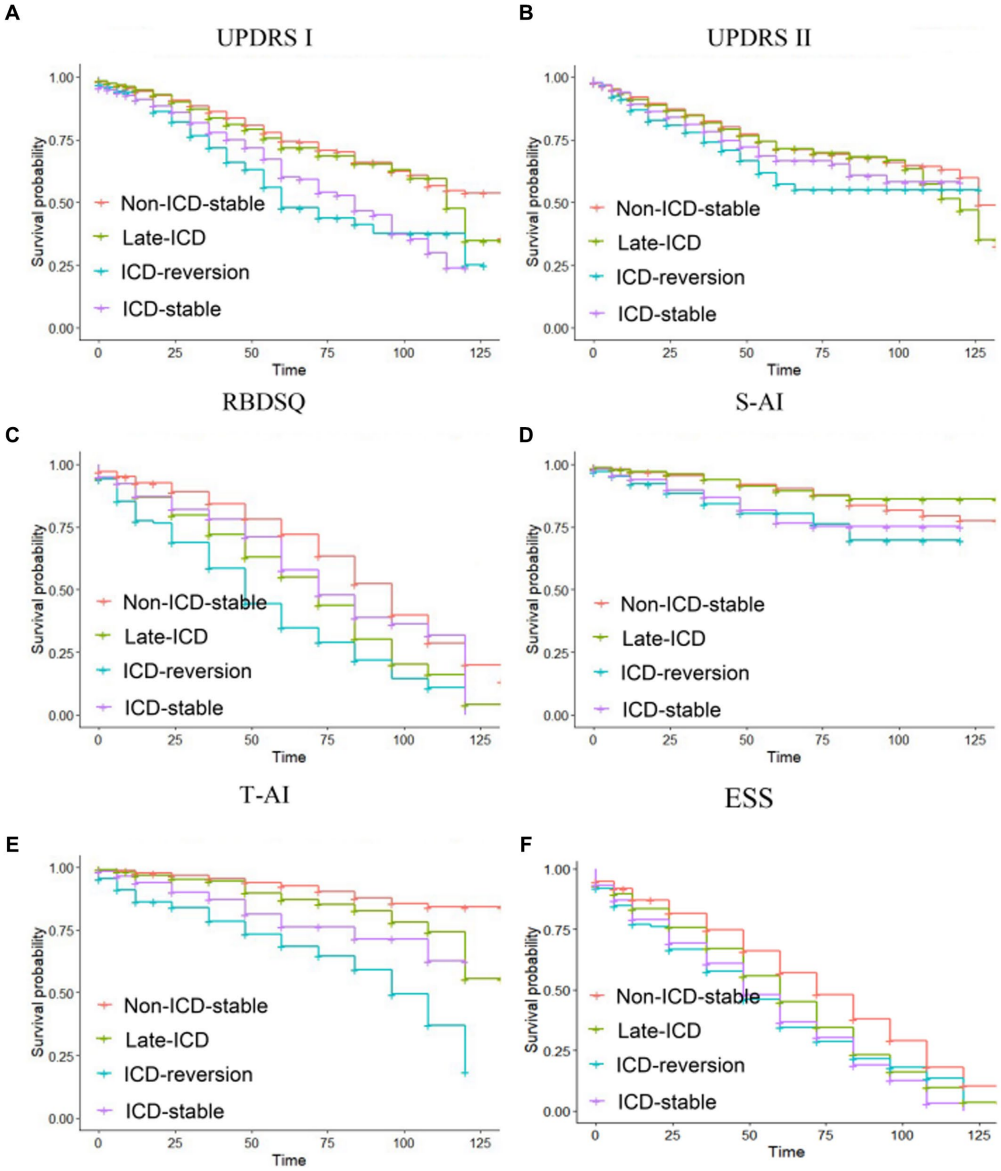
Kaplan–Meier progression-free survival curves depending on ICD evolution patterns. Time from baseline to (A) a zero-point increase in MDS-UPDRS part I, (B) a 15-point increase in MDS-UPDRS part II, (C) a RBDSQ <5, (D) a S-AI scaled score (40–49 years old: male <51, female <53; more than 50 years old: male <55, female <58), (E) a T-AI scaled score (40–49 years old: male <55, female <58; more than 50 years old: male <52, female <47), and (F) an ESS < 7. Ticks indicate censoring events.

The Kaplan–Meier analysis was used to determine whether different patterns of ICD evolution are associated with the progression of non-motor function in PD. For the increase of 0 points in the MDS-UPDRS Part I score, the PFS time in the ICD-reversion group was shorter than that in the non-ICD-stable group and the late-ICD group (Figure 2A): 71.4 vs. 99 months (*p* = 0.000 < 0.05) and 71.4 vs. 98.5 months (*p* = 0.000 < 0.05). The PFS time in the ICD-stable group was also shorter than that in the non-ICD-stable and late-ICD groups: 82.1 vs. 99 months (*p* = 0.000 < 0.05) and 82.1 vs. 98.5 months (*p* = 0.000 < 0.05).

For the 15-point increase in MDS-UPDRS II score, the progression-free survival (PFS) of the non-ICD-stable group was longer than that in the late-ICD group, ICD-reversion group, and ICD-stable group (Figure 2B): 123 vs. 114 months (*p* = 0.0004 < 0.05), 123 vs. 112 months (*p* = 0.000 < 0.05), 123 vs. 113 months (*p* = 0.0005 < 0.05). There is a tendency for the ICD-reversion group to have a shorter survival period for progression. There were no statistical significance comparisons with other groups.

For RBDSQ <5, the progression-free survival of the non-ICD-stable group was longer than that in the late-ICD, ICD-reversion, and ICD-stable groups (Figure 2C): 86 vs. 67.7 months (*p* = 0.000 < 0.05), 86 vs. 53.9 months (*p* = 0.000 < 0.05), 86 vs. 74.9 months (*p* = 0.0015 <  0.05). The PFS time in the ICD-reversion group was shorter than in the late-ICD and ICD-stable groups: 53.9 vs. 67.7 months (*p* = 0.016 < 0.05) and 53.9 vs. 74.9 months (*p* = 0.000 < 0.05).

For the S-AI scaled score, the PFS time in the ICD-reversion group was shorter than that in the non-ICD-stable group and the late-ICD group (Figure 2D): 105 vs. 117 months (*p* = 0.002 < 0.05), 105 vs. 120 months (*p* = 0.0039 < 0.05). The PFS time in the ICD-stable group was shorter than in the non-ICD-stable and late-ICD groups: 108 vs. 117 months (*p* = 0.0002 < 0.05) and 108 vs. 120 months (*p* = 0.0023 < 0.05).

For the T-AI scaled score, the PFS time comparison of the four groups was significant: the ICD-reversion group < the ICD-stable group < the late-ICD group < the non-ICD-stable group (85.5 months <104.4 months <112.5 months <120.8 months, and the *p* < 0.05; Figure 2E). For the ESS score, compared with the ICD reversal group, the ICD stabilization group, and the ICD late-onset group, the PFS time in the non-ICD group was longer (72.4 vs. 54.2 months, 72.4 vs. 53.9 months, and 72.4 vs. 60.7 months; all *p* < 0.05; Figure 2F).

### Kaplan–Meier estimates for HR in different ICD evolution patterns on non-motor progression

As shown in Table 2, we found that the ICD reversion group was associated with the progression of NMS, including an increase of 0 points in the MDS-UPDRS Part I score and 15 points in MDS-UPDRS II, REM sleep disorders, S-AI, T-AI, and ESS (HR = 2.135, 95% CI 1.892–2.379, *p* = 0.000; HR = 1.499, 95% CI 1.236–1.762, *p* = 0.003; HR = 1.499, 95% CI 1.236–1.762, *p* = 0.003 2.774, 95% CI 2.535–3.013, *p* = 0.000; HR = 2.104, 95% CI 1.628–2.58, *p* = 0.002; HR = 4.997, 95% Cl 4.605–5.388, *p* = 0.000; and HR = 1.745, 95% Cl 1.551–1.978, *p* < 0.001). The ICD stable group was also associated with the progression of NMS, including an increase of 0 points in MDS-UPDRS Part I score and 15 points in MDS-UPDRS II, REM sleep disorders, S-AI, T-AI, and ESS (HR = 1.811, 95% CI 1.625–1.997, *p* = 0.000; HR = 1.294, 95% CI 1.093–1.496, *p* = 0.012; HR = 1.349, 95% CI 1.132–1.566, *p* = 0.007; HR = 2.693, 95% CI 1.35–3.035, *p* = 0.000; HR = 3.567, 95% CI 3.217–3.916, *p* = 0.000; and HR = 1.693, 95% CI 1.53–1.857, *p* < 0.001). The late ICD group may affect REM sleep disorders, T-AI, and ESS (HR = 1.732, 95% CI 1.57–1.894, *p* = 0.000; HR = 1.821, 95% CI 1.454–2.17, *p* = 0.001; and HR = 1.397, 95% CI 1.253–1.54, *p* < 0.001).

**Table 2:** Kaplan–Meier estimates for HR in different ICD evolution patterns of non-motor progression.

Cox	Late-ICD	*p*-value	ICD-reversion	*p*-value	ICD-stable	*p*-value
	HR (95% CI)		HR (95% CI)		HR (95% CI)	
MDS-UPDRS I	1.117 (0.928–1.305)	0.252	**2.135 (1.892–2.379)**	**0.000**	**1.811 (1.625–1.997)**	**0.000**
MDS-UPDRS II	1.092 (0.91–1.274)	0.344	**1.499 (1.236–1.762)**	**0.003**	**1.294 (1.093–1.496)**	**0.012**
RBDSQ	**1.732 (1.57–1.894)**	**0.000**	**2.774 (2.535–3.013)**	**0.000**	**1.349 (1.132–1.566)**	**0.007**
S-AI	1.054 (0.664–1.444)	0.790	**2.104 (1.628–2.58)**	**0.002**	**2.693 (2.35–3.035)**	**0.000**
T-AI	**1.812 (1.454–2.17)**	**0.001**	**4.997 (4.605–5.388)**	**0.000**	**3.567 (3.217–3.916)**	**0.000**
ESS	**1.397 (1.253–1.54)**	**8E-16**	**1.745 (1.511–1.978)**	**8E-16**	**1.693 (1.53–1.857)**	**8E-16**

Cox regression, compared with the different ICD evolution patterns. Significant *p*-values are shown in bold (*p* < 0.05/3 = 0.017). ESS, Epworth Sleepiness Scale; MDS-UPDRS, Movement Disorder Society Unified Parkinson’s Disease Rating Scale; RBDSQ, REM Sleep Behavior Disorder Screening Questionnaire; S-AI, State Anxiety Inventory; T-AI, Trait Anxiety Inventory.

### Patterns of white matter microstructure change with different ICD evolution patterns at baseline in PD patients

We hypothesized that in PD patients with different patterns of ICD evolution, brain regions associated with non-motion had significant lesions at baseline. Therefore, 49 patients with non-ICD-stable, 16 with late-ICD, 6 with ICD-stable, and 4 with ICD-reversion were analyzed through whole-brain diffusion tensor imaging (DTI). Table 1 shows no significant differences between the four groups regarding age, sex, years of schooling, or length. Additionally, there were no statistically significant differences in mean Levodopa Equivalent Daily Dose during follow-up between the four groups (Supplementary Table S1).

**Figure 3:**
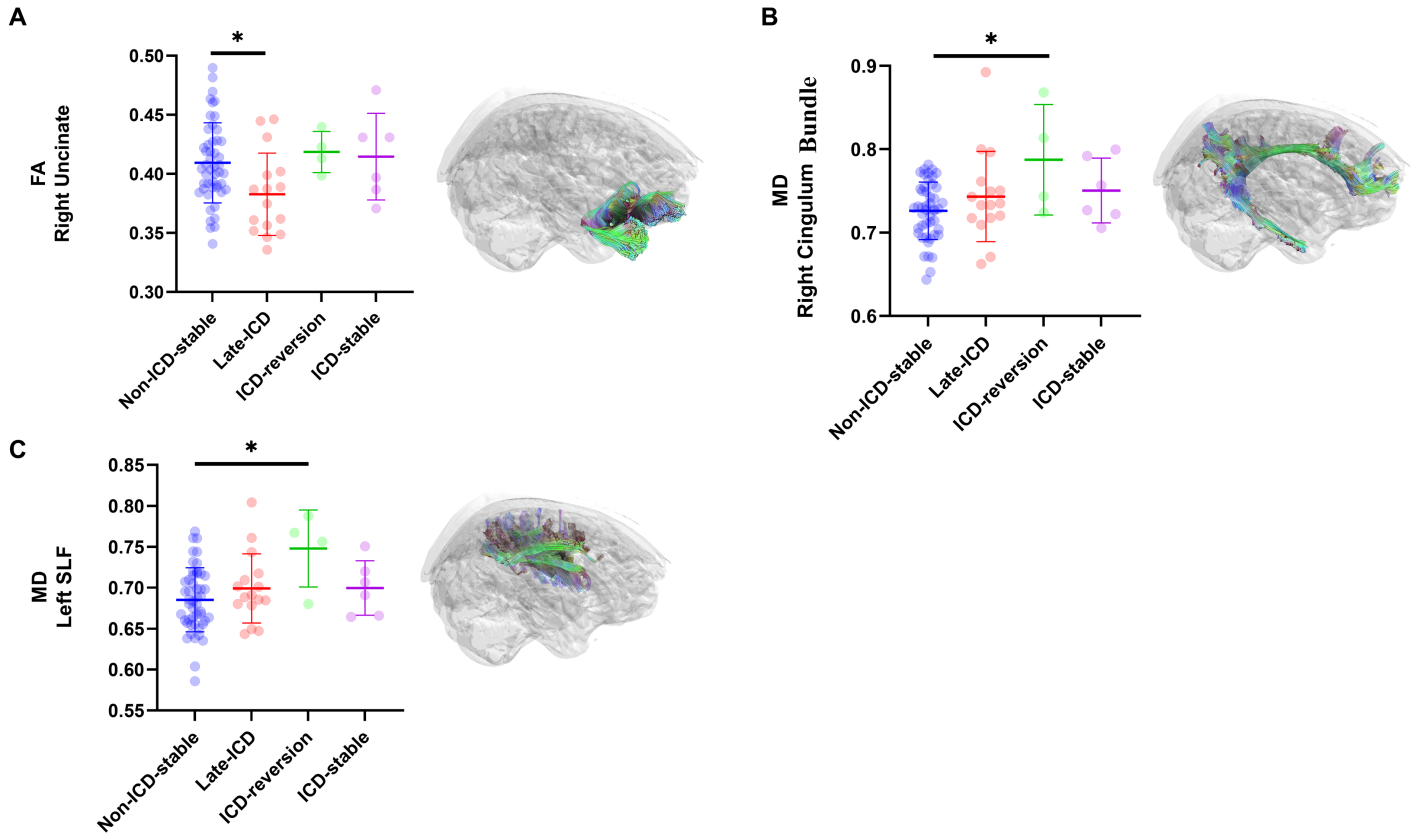
DTI differences among ICD evolution patterns. Post hoc ROI analysis in (A) the right uncinate for FA (*p* < 0.05), (B) the right Cingulum Bundle for MD (*p* < 0.05), (C) the left SLF for MD (*p* < 0.05), Bonferroni correction, error bars represent SD. *p < 0.05.

PD patients with different ICD evolution patterns have fractional anisotropy (FA) evolution patterns, mainly located on the right uncinate. The FA values of the right uncinate decreased in the late-ICD group compared with the non-ICD-stable group, the ICD-reversion group, and the ICD-stable group (FA values were 0.383 ± 0.018, 0.409 ± 0.01, 0.419 ± 0.028, and 0.415 ± 0.039, respectively; *p* = 0.037 < 0.05; Figure 3A). The FA values were positively correlated with axonal integrity; this shows that the shaft breakout of the late-ICD group was the most serious.

PD patients with different patterns of ICD evolution also have different patterns of mean diffusion (MD) change, mainly located in the right cingulum bundle and the left superior longitudinal fasciculi (SLF). In the right cingulum bundle, the ICD-reversion group had higher MD values than the non-ICD-stable, late-ICD, and ICD-stable groups (MD values were 0.787 ± 0.073, 0.726 ± 0.01, 0.743 ± 0.029, and 0.750 ± 0.04, respectively; *p* = 0.024 < 0.05; Figure 3B). In the left SLF, the ICD-reversion group had higher MD values than the non-ICD-stable, late-ICD, and ICD-stable groups (MD values were 0.748 ± 0.075, 0.685 ± 0.011, 0.699 ± 0.022, and 0.700 ± 0.027, respectively; *p* = 0.024 < 0.05; Figure 3C), which shows that the cellular edema in the ICD-reversion group was the most severe.

## Discussion

A longitudinal study of ICD in *de novo* PD patients was conducted. First, we found that ICD in PD fluctuated over time and could be divided into non-ICD-stable, late-ICD, ICD-stable, and ICD-reversion patterns. Second, we showed that ICD evolution patterns are associated with non-motor impairment in PD patients. ICD-reversion patients showed the fastest non-motor disease progression, whereas non-ICD-stable patients showed relatively mild disease progression. Third, we found that PD patients with different ICD evolution patterns had different changes in white matter microstructure at the onset of the disease. Interestingly, those relevant brain regions are involved in ICD and non-motor functions. Based on these results, ICD evolution patterns in PD may have prognostic value.

Previous longitudinal studies have found that the overall prevalence of ICD changes as the course of PD progresses. An identical incidence of 18% has been found in the normal population and patients with newly diagnosed drug-naive PD (Yoo et al., 2015). Follow-up studies have found that patients with idiopathic PD are more likely to have ICD than healthy controls and patients with newly diagnosed untreated PD (Antonini et al., 2011), with an incidence of up to 25% (Bostwick et al., 2009), suggesting that patients with PD who do not have an ICD at the time of onset may develop an ICD. Our study found that in PD patients, the incidence of ICD was 26.16% as of the fourth year of follow-up; longitudinal data show that ICD symptoms in PD patients do not always remain stable. In our study, as of year 4 of follow-up, 14.6% of PD patients without ICD at baseline developed ICD symptoms, while 5.6% of ICD patients no longer had ICD symptoms. The number of patients with PD-ICD has increased. In a recent 4-year prospective cohort study of DA therapy, the incidence of ICD was 39% (Bastiaens et al., 2013).

The incidence of ICD in PD patients treated with DA has been shown in previous studies, fluctuating between 15 and 40% (Garcia-Ruiz et al., 2014; Weintraub et al., 2015). We reported the disappearance of ICD symptoms in 5.6% of patients with ICD, and the reversibility of ICD and related behaviors after dopamine agonist discontinuation has been fully demonstrated in a few previous studies (Bostwick et al., 2009). According to this study, we observed that ICDs vary with the course of PD and that ICDs may persist or reverse their disappearance. This difference may reflect the heterogeneity of PD, with different subtypes. Therefore, the evolution model of ICD may have predictive value.

Second, we determined the progress of PD patients with ICD evolution patterns and NMSs. Specifically, non-ICD-stable patients showed relatively mild disease progression, whereas ICD-reversion patients showed the fastest NMS progression. This study focuses on the link between the ICD change course and PD progress. A previous study showed that compared with non-ICD in PD patients, patients with ICD have more than NMS, consistent with the results of our study. Further, we analyzed the effects of each group on NMS of daily life (MDS-UPDRS Part I), the severity was as follows: the ICD-reversion group > the ICD-stable group > the late-ICD group≈ the non-ICD-stable group. Here, we also found that the evolution patterns of ICD correlated with different rates of progression of NMS, generally manifested as the following trends: the ICD-reversion group > the ICD-stable group > the late-ICD group > the non-ICD-stable group.

Previous reports have shown that patients with ICD have more frequent rapid eye movement sleep behavior disorders (RBDs) than those without ICD (Fantini et al., 2015). Those results are consistent with our study. The effects of each group on rapid eye movement behavior disorder (RBDSQ-5) were further analyzed, and the severity was as follows: the ICD-reversion group > the ICD-stable group > the late-ICD group > the non-ICD-stable group.

Consistent with our results, patients with ICD have more obvious anxiety symptoms than those without ICD (Voon et al., 2011). We further analyzed different groups to determine the T-AI score and S-AI score. Among them, the S-AI score affects the severity of the sequence: the ICD-reversion group ≈ the ICD-stable group > the late-ICD group ≈, and the non-ICD-stable group. The T-AI-score affects the severity of the sequence: the ICD-reversion group > the ICD-stable group > the late-ICD group > the non-ICD-stable group.

Siri’s study has shown that compared with patients with ICD and those without ICD, there are no differences in cognitive function in PD patients, consistent with our results (Siri et al., 2010). However, there is a paucity of research on the role of PD patients with and without ICD in motor symptoms had no effect. However, our study also found that the ICD-stable group had a better experience of exercise in daily life compared to the non-ICD-stable group. Moreover, the effect of severity on everyday life movement symptoms (MDS-UPDRS part II) in different groups was as follows: the ICD-reversion group > the ICD-stable group ≈ the late-ICD group > the non-ICD-stable group.

Previous studies on PFS using the Epworth Sleepiness Scale (ESS) scores of patients with PD-ICD are rare. For the ESS score, our study found that compared with the ICD-reversion group, the ICD-stable group, and the late-ICD, the PFS time in the non-ICD-stable was longer (72.4 vs. 54.2 months, 72.4 vs. 53.9 months, and 72.4 vs. 60.7 months; all *p* < 0.05). Above all, the two types with the worst prognosis (the ICD-reversion and ICD-stable groups) constitute a baseline ICD group. Therefore, ICD evolution patterns and associations between PD progress. Furthermore, we found that the ICD-reversion group was more relevant to the progress of RBD, anxiety symptoms, and motor symptoms.

Third, we demonstrated that the alteration patterns of white matter microstructure differed among PD patients whose ICD evolution patterns differed. Interestingly, both ICD and non-motor functions are associated with relevant brain regions. These findings suggest that the ICD evolution pattern is potentially prognostic in PD.

Multiple imaging studies have found that changes in brain structure, function, and metabolism prior to drug therapy in PD patients increase the risk of developing an ICD (Ray and Strafella, 2013; Aracil-Bolanos and Strafella, 2016; Mojtahed Zadeh et al., 2018; Santangelo et al., 2019; Baagil et al., 2023; Hernadi et al., 2023). Yoo et al. (2015), using DTI technology, found that in the anterior corpus callosum, right internal capsule posterior limbs, right posterior cingulum, and right thalamic radiations, FA levels in the PD-ICD were significantly higher than in PD-non-ICD patients (corrected *p* < 0.05). In our study, the PD-ICD group had a higher FA trend than the non-ICD group in the right uncinate. The late-ICD group had the lowest FA (*p* < 0.05).

Previous research has suggested that the uncinate fasciculus plays a hypothetical role in several psychiatric disorders, including episodic memory, language, and social–emotional processing (Von Der Heide et al., 2013). Therefore, for PD patients, whether the change in the right uncinate FA value is related to the development of ICD and whether it is a predictor of future ICD needs to be further studied.

Based on previous studies, cognitive dysfunction in specific areas was related to damage to certain tract profiles, including the posteromedial component of the right cingulum bundle, the posterior portion of the left SLF, the bilateral anterior thalamic radiation, and the occipital lobe portion of the callosum forceps major (Huang et al., 2020). Increased MD is often associated with a loss of microstructural integrity (Aslan and McCarty, 2013). In our study, in the right cingulum bundle and left SLF, MD values were higher in the ICD-reversion group than in the other three groups (*p* < 0.05). According to the results, the ICD-reversion group had the most serious microstructural damage in the right cingulum bundle and left SLF, which may explain the factors that progressed most rapidly to non-motor function in the ICD-reversion group. This can explain the clinical features of different ICD evolutionary patterns.

### Limitations

First, the ICD diagnosis is based on an impulse and obsessive-compulsive disorder PD screening questionnaire (QUIP) (Weintraub et al., 2009). The QUIP was designed and validated as a screening tool, not a diagnostic or rating tool (Weintraub et al., 2009). In combination with clinical manifestations in patients with final confirmation, clinical doctors must improve clinical diagnosis accuracy. In assessing ICD and other compulsive behaviors during PD, we used an “anytime” time frame to avoid recall bias (Weintraub et al., 2009). However, in recent clinical studies, questionnaire-based evaluation is still widely used because it is relatively time-saving and practical (Marin-Lahoz et al., 2022). The QUIP, a self-administered questionnaire for recognizing ICD in Parkinson’s patients, is a “gold standard” diagnosis using formal diagnostic criteria with good discriminant validity (Weintraub et al., 2009).

Second, the ICD reverse group sample size is relatively limited (18 patients). Especially MRI data, ICD-reversion group only included four patients. The underlying mechanisms can be better examined with larger cohorts to validate our preliminary findings. Large longitudinal studies are needed to validate the prognostic value of ICD evolutionary models, an important issue that can significantly impact the outcome. It could be improved by discussing the clinical implications of the observed ICD evolution patterns, particularly regarding patient management and treatment strategies. Finally, because we focused exclusively on *de novo* PD patients, medication and dosage variations may interfere with ICD and PD symptoms.

In conclusion, based on our study, four early ICD evolutions are identified in *de novo* PD, with different prognoses and brain white matter microstructural damage patterns, and may predict motor progression and cognitive decline in PD patients to develop precise intervention strategies as early as possible.

## Methods

### Study design and participants

Our data is from the Parkinson’s Progression Markers Initiative (PPMI) database; PPMI is a multicenter study of early-stage PD subjects in longitudinal studies, and detailed objectives and methods for this study have previously been published (Evans et al., 2019).

The PPMI database was downloaded in March 2022. PD patients meeting both criteria were included in this study: (1) a baseline age of 40 years for men or women, and (2) clinical assessment data were available. After enrollment, participants were followed up every 3 months for the first year and followed every 6 months after that. Patients were excluded if they have: (1) follow-up for less than 4 years; (2) DBS surgery during follow-up; and (3) during the follow-up period, they were diagnosed with multiple system atrophy, essential tremor, and dementia with Lewy bodies.

### Ethical approval

The PPMI study has been registered with ClinicalTrials.gov under registration number NCT01141023. Each participating site of the study was approved by the Human Experimentation Ethics Standards Committee, and participants provided informed consent to participate.

### Assessment and classification of ICD transformation

#### The diagnosis of ICD was assessed using the QUIP-rating scale

To standardize the clinical diagnosis and research of PD-ICD, the International Association of Movement Disorders recommends using QUIP and the QUIP-RS for the screening, classification, and evaluation of the occurrence of PD-ICD after systematically evaluating the 50 reported ICD evaluation and grading scales (Von Der Heide et al., 2013). The QUIP scale is a quick and easy PD-ICD rating scale designed by scholars such as Weintraub of the University of Pennsylvania School of Medicine in 2009. Based on the previous ICD screening scale, Professor Weintraub followed the diagnostic criteria and clinical features of the revised fifth edition of the American Diagnostic and Statistical Manual of Psychiatry and divided the QUIP scale into three parts: (1) five questions for the four most common ICDs of PD; (2) five questions for compulsive impulse behaviors in PD; and (3) five questions for dopamine dysregulation syndrome (DDS). Under the premise of ensuring more than 80% sensitivity and specificity, the positive answer to two questions ≥ pathological gambling is positive on the QUIP scale; impulse shopping ≥ positive answer to one question; compulsive eating ≥ positive answer to two questions; and compulsive sexual behavior ≥ positive answer to one question. The QUIP scale requires clinicians to make final confirmation based on the patient’s clinical manifestations to improve the accuracy of clinical diagnosis. The sensitivity and specificity of the QUIP scale have been verified in many countries and regions. QUIP-RS is a scale developed by QUIP to measure the severity of PD-ICD, involving 28 questions on pathological gambling, impulsive shopping, compulsive eating, compulsive sexual behavior, stereotypic movements, special hobbies, and the occurrence of DDS within 1 month. Each item is evaluated on a five-point scale, and its score ranges from 0 (never) to 4 points (very), with a total score of 0–112 points. The higher the score, the heavier the severity of the disease, under the premise of ensuring more than 90% sensitivity and specificity. On the QUIP-RS scale, pathological gambling ≥6 points is higher. Impulse shopping ≥8 is considered more severe. Compulsive eating ≥ a score of 7 is considered more severe. Compulsive sexual behavior ≥ a score of 8 is considered more severe (Evans et al., 2019).

During the fourth year of follow-up, the pattern of ICD evolution was gaged according to the ICD status during the baseline year. PD patients were defined as (1) ICD with a positive QUIP-RS score at baseline and subsequent visits or (2) non-ICD with a negative QUIP-RS score at baseline and subsequent visits.

Patients were excluded in the first year of follow-up if they did not achieve a stable ICD status. Patients were divided into four groups based on symptom fluctuations in the fourth year of follow-up. (1) Non-ICD stable: patients were ICD-free at baseline and throughout the 4-year monitoring period. (2) Late ICD: patients were ICD-free at baseline but developed an ICD during the 4-year follow-up period. (3) Stable ICD: patients with ICD symptoms during the 4-year follow-up period. (4) ICD recovery: the ICD symptoms were present at baseline but disappeared during the 4-year follow-up period.

### Clinical assessments

During follow-up, the investigators assessed the patient for multiple motor and NMSs to understand disease progression, including the Movement Disorders Association in conjunction with the Parkinson’s Disease Rating Scale (MDS-UPDRS) Parts I (non-motor experiences of daily living), II (motor experiences of daily living), III (motor examination), and Hoehn-Yahr (H&Y). NMSs were evaluated, including the ESS scaled score, RBDSQ scaled score, GDS scaled score, S-AI scaled score, and T-AI scaled score.

The global and domain-specific cognitive status of the participants was assessed with neuropsychological tests, including the Hopkins Verbal Learning Test-Revised (HVLT-R) for verbal memory, the Montreal Cognitive Assessment (MoCA) for global cognition; the Semantic Fluency Test animal category for verbal fluency; the Judgment of Line Orientation for visuospatial ability; the Letter-Number Sequencing for working memory; and the Symbol Digit Modalities Test for executive function. As previously described, the performance on these assessments was converted to t-scores or scaled scores (Wyman-Chick et al., 2018).

### Outcomes

Criteria for the progression of NMSs are as follows:

1. Criteria for sleep progression: RBDSQ <5.
2. The standard for MDS-UPDRS Part I is MDS-UPDRS Part I < 0.
3. MDS-UPDRS Part II < 15.

The State–Trait Anxiety Scale (STAI) is applied to determine whether a client has a transient anxiety attack and state or more stable personality traits with chronic anxiety levels. It is a self-report scale (10.1037/h0020743). In males aged 40–49, a cutoff of 51 or greater is used to define State-Anxiety. In females aged 40–49, a cutoff of 53 or greater is used to define State-Anxiety. In males aged 40–49, a 55 or greater cutoff is used to define Trait-Anxiety. In females aged 40–49, a cutoff of 58 or greater is used to define Trait-Anxiety. In males aged 50 or more, a cutoff of 55 or greater is used to define State-Anxiety. In females aged 50 or more, a cutoff of 58 or greater defines State-Anxiety. In males aged 50 or more, a cutoff of 52 or greater is used to define Trait-Anxiety. In females aged 50 or more, a cutoff of 47 or greater is used to define Trait-Anxiety.

### Neuroimaging and DTI analysis

During the 6 months of follow-up, some patients underwent high-resolution three-dimensional T1-weighted MRI scans, which ruled out significant abnormalities due to excessive head movement artifacts. A total of 75 patients met the requirements.

In addition to the above details, the PPMI MRI operation manual contains additional information. DTI analysis was performed following the previous publication (Sun et al., 2015). Briefly, we used FSL 6.0.5 (FMRIB Software Library, FMRIB, Oxford, United Kingdom). An automated fiber quantification (AFQ) v0.1 program is available at https://github.com/jyeatman/AFQ (Yeatman et al., 2012). The diffusion images were preprocessed using FSL. The preprocessing steps, including B0, were registered with a DWI image, head motion correction, and an exclusion of non-brain tissue. After preprocessing, we used the AFQ to perform fiber tracking and tract segmentation. A detailed description of the method of neuroimaging processing is shown in Supplementary File S1.

A structured array with tensor-based measures was returned for each group of 20 tracts. We focused specifically on FA, MD, axial diffusivity (AD), and radial diffusivity (RD) using 100 nodes per tract delineated: left and right thalamic radiations, minor of the corpus callosum and forceps major, left and right inferior frontal-occipital, superior longitudinal, inferior longitudinal, corticospinal tract, arcuate and uncinate fasciculi, and cingulum.

### Statistical analysis

Analysis of statistical results using SPSS 25 (IBM Corp., Armonk, NY). Continuous variables are expressed as mean ± standard deviation and categorical variables are expressed as quantities (percentages). Chi-square tests are used to find associations between categorical variables. One-way analysis of variance (ANOVA) is used for continuous variable calculations.

Kruskal–Wallis with Bonferroni correction was used for multiple comparisons. The chi-square or Fisher’s exact test was used with categorical data to represent and compare frequencies and percentages. PFS was calculated using the Kaplan–Meier method, and a log-rank test compared survival rates. A Cox proportional hazard model was used to estimate the risk ratio (HR).

A *p*-value <0.05 was a considered statistically significant. For DTI analysis, we extracted the average FA, MD, AD, and RD values of different fiber celluloses. Our study generated 1,000 bootstrap samples and applied ANOVA to each bootstrap sample. After testing for the homogeneity of variances, Bonferroni was used to correct t-values for multiple comparisons; otherwise, Dunnett’s *t*-test was used. In this instance, statistical significance was defined as a *p*-value of less than 0.001.

## Data availability statement

The datasets presented in this study can be found in online repositories. The names of the repository/repositories and accession number(s) can be found in the article/Supplementary material.

## Ethics statement

The studies involving humans were approved by The PPMI study has been registered with ClinicalTrials.gov under registration number NCT01141023. Each participating site of the study was approved by the Human Experimentation Ethics Standards Committee, and participants provided informed consent to participate. The studies were conducted in accordance with the local legislation and institutional requirements. The participants provided their written informed consent to participate in this study.

## Author contributions

LH: Conceptualization, Data curation, Formal analysis, Funding acquisition, Methodology, Project administration, Resources, Software, Writing – original draft, Writing – review & editing. CL: Conceptualization, Data curation, Formal analysis, Methodology, Project administration, Software, Writing – original draft, Writing – review & editing. FL: Conceptualization, Data curation, Formal analysis, Funding acquisition, Methodology, Project administration, Software, Writing – original draft, Writing – review & editing, LW: Investigation, Supervision, Validation, Visualization, Writing – original draft. ZL: Investigation, Supervision, Validation, Visualization, Writing – review & editing. ZC: Investigation, Supervision, Validation, Visualization, Writing – review & editing. XL: Investigation, Supervision, Validation, Visualization, Writing – review & editing. QY: Conceptualization, Data curation, Project administration, Supervision, Validation, Writing – original draft. YW: Conceptualization, Data curation, Project administration, Supervision, Validation, Writing – review & editing. GC: Conceptualization, Data curation, Project administration, Supervision, Validation, Writing – review & editing.
