# Supplementary material for: Different impulse control disorder evolution patterns and white matter microstructural damage in the progression of Parkinson’s disease
Source: Front Aging Neurosci. 2023 Dec 22;15:1260630. doi: 10.3389/fnagi.2023.1260630 (PMC10768538; doi:10.3389/fnagi.2023.1260630)
Supplement: Supplementary file 1 [file Table_3.docx]

Supplementary Table 1. Mean LEDD during follow-up in the ICD pattern.

|  | LEDD | P-value |
| --- | --- | --- |
| Non-PIGD-stable vs. Late-PIGD | 274.69±509.8 vs. 311.8±672.47 | 0.819958 |
| Non-PIGD-stable vs. PIGD-reversion | 274.69±509.8 vs. 446.52±1105.62 | 0.216859 |
| Non-PIGD-stable vs. PIGD-stable | 274.69±509.8 vs. 276.61±345.1 | 1 |
| Late-PIGD vs. PIGD-reversion | 311.8±672.47 vs. 446.52±1105.62 | 0.551912 |
| Late-PIGD vs. PIGD-stable | 311.8±672.47 vs. 276.61±345.1 | 0.897134 |
| PIGD-reversion vs. PIGD-stable | 446.52±1105.62 vs. 276.61±345.1 | 0.241282 |

LEDD, Levodopa Equivalent Daily Dose; ICD, impulse control disorder;

PIGD, postural instability and gait difficulties
